# Global Public Health Database Support to Population-Based Management of Pandemics and Global Public Health Crises, Part I: The Concept

**DOI:** 10.1017/S1049023X20001351

**Published:** 2020-10-22

**Authors:** Frederick M. Burkle, David A. Bradt, Benjamin J. Ryan

**Affiliations:** 1. Professor (Ret.) Senior Fellow and Scientist, Harvard Humanitarian Initiative, Harvard University, T.H. Chan School of Public Health, Cambridge, Massachusetts USA; 2. Global Scholar, Woodrow Wilson International Center for Scholars, Washington, DC USA; 3. Dept of International Health, Johns Hopkins School of Public Health, Baltimore, Maryland USA; 4. Clinical Associate Professor, Department of Environmental Science, Baylor University, Waco, Texas USA

**Keywords:** global public health, pandemics, population-based management, public health, triage

## Abstract

This two-part article examines the global public health (GPH) information system deficits emerging in the coronavirus disease 2019 (COVID-19) pandemic. It surveys past, missed opportunities for public health (PH) information system and operational improvements, examines current megatrend changes to information management, and describes a new multi-disciplinary model for population-based management (PBM) supported by a GPH Database applicable to pandemics and GPH crises.

## Introduction

The novel severe acute respiratory syndrome (SARS)-CoV-2 virus (coronavirus disease 2019 [COVID-19]) is the first coronavirus to drive a pandemic. COVID-19 is a unique and challenging virus to manage due to the high person-to-person transmissibility, incubation period, and potential for asymptomatic and symptomatic people to spread the disease. While global efforts are underway to develop new clinical therapies, vaccines, and safety measures, there are rising efforts to improve underlying public health (PH) systems. In February 2020, the World Health Organization (WHO; Geneva, Switzerland) convened a global forum on research and innovation for COVID-19 to identify immediate research actions, mid-term and long-term priorities, and cross-cutting research priorities. Biomedical priorities dominated the agenda, underscoring multi-sectoral knowledge gaps and challenges that must be addressed for pandemic health management. Less attention was given to information management and decision support systems.

External to WHO, there have been calls arising for improvements in many niches of PH and decision support systems. These include calls for increased transparency in the deliberations of WHO Emergency Committees responsible for making recommendations on PH events of international concern,^1^ call for a new National Infectious Disease Forecasting Center, and a new National Center for Pandemic and Disaster Nursing Research.^2^ There are also calls for epidemic management at local levels, to include testing and tracing action plans; to benchmark disease incidence and link it to community risk levels; and to develop checklists measuring health systems capacities and capabilities.^3-5^ Many of these recommendations emerge from non-operational actors—academic institutions, foundations, and universities. Among these disparate recommendations, there has been little attention to operational issues surrounding PH decision making—maintenance of relevant databases, identification of sentinel event outliers and occult trends, and urgency of decision making for vulnerable populations.^3,6,7^

## Missed Historical Opportunities

Recent transnational epidemics have exposed recurring weaknesses in the current state of pandemic management. In early 2003, the SARS-CoV epidemic affected over 8,000 people in 26 countries resulting in a fatality rate of 15%, but by late July, the outbreak was contained.^7^ Despite the rapid containment, non-compliance with necessary PH decisions, often politically motivated, ravaged Hong Kong, Taiwan, and Singapore. The outbreak resulted in serious difficulties in obtaining public cooperation, with large-scale failures to cooperate with epidemiological contact tracing, mass disobedience of quarantine orders, and hospitals concealing SARS cases. These events globally led to the 2005 revision of International Health Regulations (IHR) coming into force in 2007. In 2007, the WHO recommended population-based simulations as an important source of knowledge when planning PH responses, but again, this too came under scrutiny by nations that it did not take into account the behavior of people or to multiple changes in a county’s precondition status, which varied widely. The WHO concluded that their population-based simulations used in training failed to support the interaction between microbiological, epidemiological, and societal progressions during a pandemic.^8^ In the spring of 2009, a novel influenza A (H1N1) virus pandemic hit, the first in 40 years. World-wide, 151,700-575,400 people died, and in the United States (US) alone, there were over 60 million cases and more than 12,000 deaths.^9^ But with early approval of a vaccine that year, the WHO declared the pandemic ended by early fall.^10^ Once again, there were major challenges to the authority of the IHR and global public health (GPH) governance. The WHO warned in 2009 that few PH surveillance systems could either detect pandemic outbreaks or warn relevant agencies and the public.

Five years later, by 2014, 48 member states had no documented progress on their obligations under IHR and 81 member states requested a further two-year extension to fulfill their obligations. Some disgruntled member states initiated the Global Health Security Agenda in 2014 as a partnership of nations, international organizations, and nongovernmental organizations to elevate global health security as a global priority.^11^

The West Africa Ebola epidemic of 2014-2015 then revealed gross deficiencies in epidemic preparedness of countries in the region, as well as emergency management throughout the WHO. This led to the standup of the United Nation’s (UN) first emergency health mission, the UN Mission for Ebola Emergency Response (UNMEER), from September 2014 through July 2015. An emergency special session of the Executive Board in January 2015 and the final report of the external Ebola Interim Assessment Panel (Stocking Report) in July 2015 helped precipitate reforms deemed critical for the WHO’s continued role in emergencies. The WHO launched a new Health Emergencies Program in 2016 under a newly recruited Deputy Director-General. The WHO also convened a broad coalition of experts in 2015 to produce a Research and Development Blueprint for Action to Prevent Epidemics. The 2015 blueprint explicitly cited a need for urgent action for highly pathogenic coronaviruses relevant to humans, including SARS and Middle East respiratory syndrome (MERS). Coronaviruses have remained on the blueprint’s annual list of priority pathogens ever since. Nonetheless, there were no significant international coronavirus vaccine development efforts until the advent of COVID-19, yet it was painfully clear that a wider and more prolonged pandemic would require much greater management capacity than the existing PH resources could possibly provide.

In 2010, rare academic courses were offered that covered the lessons learned from both SARS and H1N1, and for the first time, introduced the concept of population-based management (PBM) that would require a much stronger PH workforce and leadership.^12,13^ More speculative “but what if?” questions led, especially by physicians and nurses, to concerns regarding triage management and care of patients who exceeded available resources. Several studies were published that dealt with population-based triage management of casualties, and for the first time, introduced the category of “unsustainable” or “removed” which required collective decision making by a proposed PBM team (PBMT) who knew what resources were available and what was not. The PBMT would dictate the necessary triage decision to the individual health providers, relieving them of the responsibility of making these uncomfortable but real decisions alone.^14^ Years later, these same PBM triage studies became the nidus of a major 2016 desk-top exercise called Operation Cygnus by Public Health England (London, UK), which was designed to manage a major influenza pandemic; again in 2017, Scotland used the same PBMT triage studies in their Operation Isis pandemic trial. However, any potential lessons learned in PBM ultimately failed to be brought forward to guide the 2020 COVID-19 response, resulting in “serious shortcomings in preparation against a major PH emergency.”^15^

The rapid 2020 spread of COVID-19 led to singularly independent decision-making efforts of nations and jurisdictions within nations. Under the decentralized response system in the US, 50 States, subsuming 3,143 counties or country-equivalents with autonomous PH responsibilities, demonstrated variable approaches to data management, COVID-19 testing strategies, quarantine rules, and mitigation measures. Governors occasionally issued state-wide mandates, which were challenged by officials in subordinate jurisdictions. Ultimately, states competed among themselves for scarce hospital resources for the sickest patients.^16^ On March 29, 2020, US Vice President Pence sent a letter to hospital administrators across the country requesting daily data reports be sent to the government’s Health and Human Services (HHS; Washington, DC USA) on testing, capacity and utilization, and patient flows relating to COVID-19.^17^ While this provided rapid situational awareness for leaders and decision makers, it bypassed the Centers for Disease Control and Prevention (CDC; Atlanta, Georgia USA), the established major locus of control for aggregating health information and decision making in a pandemic.^18^ Meanwhile, the CDC maintained technical support agreements with States that depended on State requests for formal CDC engagement.

Unlike other pandemics, COVID-19 and how it has been managed has brought many PH experts to conclude the response and the outcomes were predictable and preventable.^19^ Also, WHO warns “there’s no going back to ‘old normal,’” asserting PH decisions are the “difference between life and death…”^20^ A key challenge is that in many nations, including the US, crises are addressed “through a litany of programs housed in several different agencies and funded by different buckets.” The result is often a resistance towards taking a holistic approach to PH security and preparedness.^21,22^ Hotez, Dean of Tropical Medicine at Baylor University (Waco, Texas USA), claims the coronavirus campaign is driven by a “White House disinformation campaign.”^23^

The rapid 2020 spread of COVID-19 began to question singular, independent decision-making efforts as the response model used in the States of the US. In a short time, an “every man for himself” mentality took over. If a state does not request CDC assistance, they won’t go in. All 50 US States demonstrated quite different approaches to health care, data analysis, and decision making, resulting in unprecedented opportunities for increasing economic and political interpretation and control of health care management at both the State and Federal levels, leaving a trail of unmet PH leadership opportunities and incomplete vital social distancing strategies.^16^ At the global level, the WHO’s surveillance network collects PH surveillance data from all countries, but nationally few data sets can either detect outbreaks or adequately warn relevant PH agencies or the public.

## Megatrends

Information management challenges in health crises such as pandemics remain predictable. These challenges involve disease screening, contact tracing, isolation and quarantine, clinical care, medical logistics, epidemic forecasting, and economic assistance to affected populations. There are numerous hurdles in managing these PH and clinical datasets—technical, administrative, financial, and ethical challenges among them. Public health information management is non-standardized across jurisdictions. Field situations are fluid. Data are perishable. Informants are mobile. Caseloads and contacts enlarge exponentially. Privacy protections are variable. The daunting task of managing such complicated datasets has led to calls for modernization of a PBM system and PH data infrastructure in conjunction with a new international infectious disease forecasting center.^2^ In this context, emerging megatrends may assist with best-practice guidance to data-driven solutions for these complexities. Selected megatrends are discussed below.

Major issues are arising on privacy, protection of personally identifiable health information, and redress for errors that implementing jurisdictions must confront. However, the application of digital technology in numerous nations with COVID-19 reveals a megatrend to be followed.

## Data Literacy

Data literacy may be defined as the ability to identify, locate, interpret, and evaluate information to ethically address a specified question or issue.^24^ Data literacy includes:

- Using data, statistics, and indicators;
- Finding or undertaking relevant research;
- Visualizing information; and
- Providing evidence for policy and PBM decision making.

A key prerequisite for any effective PH response is the availability of timely, reliable, and robust information. Public health information management is the systematic process of collecting, collating, storing, processing, verifying, and analyzing data and information, and disseminating relevant information to PH stakeholders.

For implementing organizations, the issues at hand may relate to determining the magnitude of a problem, whether an intervention is likely to work, what the possible consequences are, who should be targeted, and how something is performing. These issues are at the core of organizational service missions. Hence, data literacy and information services are inextricably related to competencies necessary for effective organizational performance. The compelling, fundamental requirement for data literacy has prompted numerous organizations to take responsibility for the education and training of their staff in these areas. Key issues include:

- Which specific methods, software applications, and tools should be used to deliver information services;
- How quickly and with what frequency of update each service should be delivered in different crisis scenarios;
- What staffing and other resources should be made available to local leaders to successfully discharge the information function; and
- Which information competencies staff should possess when deploying into the field and should, therefore, be a basis for recruitment, professional development, and performance management.

The Assessment Capacities Project (ACAPS) in the private international community; US Agency for International Development (USAID; Washington, DC USA) Data Services (DDATA) in the donor community for USAID; and Global Information Management, Assessment, and Analysis Cell (GIMAC) in the UN community are emerging as service providers and thought leaders on data literacy.^25-27^ Much more work needs to be done to mainstream technical aspects of data literacy in disaster responders.

## Evidence-Based Decision Making

Evidence-based decision making (EBDM) is a process for making decisions about a program, practice, or policy based upon best available research, contextual, and experiential evidence. This defines the duties of the regionally placed PBMTs. It requires a systematic and rational approach to researching and analyzing available evidence to inform the decision. The rationale is that evidence-based decisions produce better outcomes for stakeholders than non-evidence-based decisions. This rationale is based on:

- Validated needs assessments of the affected community which may lead to customized inputs for the population of concern;
- Information sharing with the affected community yielding clarity on what past policies/practices have or haven’t worked;
- A common sense of urgency of an issue or problem which may lead to effective resource mobilization for the interventions under consideration;
- Reduction in government expenditure by avoiding ineffective policies or programs which could be costly and time-consuming; and
- Decision making that is transparent, rational, and accountable, and thus consistent with democratic and political expectations.

Conversely, where evidence is not used as a basis for decision making, or the evidence used is not a valid reflection of unmet needs in beneficiary populations, the proposals for intervention are unlikely to produce effective outcomes and may attract negative consequences for the intended beneficiaries and donors.

Unfortunately, clinical care, PH, and disaster management have different paradigms governing their use of evidence. The disciplines have different types of questions that drive searches for evidence, different data gathering tools, different evidence hierarchies, different rules for downgrading flawed evidence, and different strategies for retrieving available evidence.^28^

All these differences complicate efforts within biomedical sciences to develop consensus conclusions and provide multi-disciplinary guidance to the public in a pandemic. Nonetheless, EBDM has become a cornerstone of best practice in biomedical disciplines over the past three decades by fostering an understanding of risks, benefits, and consequences of available choices and by improving the efficiency and effectiveness of decisional processes.^29^ Donors are increasingly requiring EBDM to merit donor funding. The challenge for disaster professionals is to understand evidence-based processes and implement them.

Evidence-based medicine was first defined in the biomedical literature in 1992 by medical practitioners at McMaster University in Ontario, Canada who sought “the conscientious, explicit, and judicious use of current best evidence in making decisions about the care of individual patients.”^30^ Evidence became organized by a hierarchy of evidence strength with a systematic review of randomized controlled trials at the apex and individual case-controlled studies and expert opinion at the bottom (Table 1).^31^

**Table 1.** Evidence Paradigms in Three Disaster Sciences

Attribute	Clinical Medicine	Public Health	International Disaster Management
Age of Current Evidence Paradigm	1992	2001	2005
Thought Leaders	Sackett, McMaster, Cochran, Oxford	CJ Murray, WHO, Academic Institutions	ERC Domestic: GOs International: IOs
Questions of Interest	PICO (well-defined)	PICO, non-PICO	non-PICO
Data Gathering Tools	SR > RCT > cohort > CCS > case series	RHA, field surveys, disease surveillance	RHA, field surveys (late)
Evidence Hierarchy	Generally, as above	No	No
Rules for Down-Grading Flawed Evidence	Per professional societies	No	No
Retrieval Strategies	Systems > synopses > syntheses > studies	Similar to EBM	HIC, sector-specific

Abbreviations: ERC, Emergency Relief Coordinator; CCS, case-control study; HIC, Humanitarian Information Center; PICO, population-intervention-comparison group-outcome; RCT, randomized controlled trial; RHA, rapid health assessment; SR, systematic review; WHO, World Health Organization.

By contrast, evidence-based PH emerged from practitioners using data-gathering tools for population-based research which differed from those in individual clinical care. Population-intervention-comparison group-outcome (PICO) as well as non-PICO questions arose, and particularly for populations in crisis, three major data-gathering tools predominated—rapid health assessments, population-based surveys, and disease surveillance.^32^ Leading practitioners understood that the strength of evidence obtained by these tools was not easily measured by the grading scales of evidence-based medicine. Moreover, they recognized the many different purposes for using evidence in PH, including strategic decision making, program implementation, and monitoring and evaluation. Hence, PH experts defined the best available evidence as the use of all available resources to provide relevant inputs for decision making.^33^

Evidence-based disaster management and humanitarian assistance (DM/HA) has arisen in disaster contexts. Non-PICO questions typically predominate in an operational environment that esteems cooperation-coordination-consensus-communication-and-assessment (C4A).^34^ Whereas evidence-based medicine affirms the ascendancy of evidence-based judgments over personal judgments regardless of how eminence-based they may be, DM/HA relies heavily on goodwill and consensus mediated by humanitarian coordinators.^35^ Overall, disaster management in this context remains fundamentally an eminence-based system.^29^

All these differences complicate efforts within biomedical sciences to develop consensus conclusions and provide multi-disciplinary guidance to the public in a pandemic. Nonetheless, EBDM has become a cornerstone of best practice in biomedical disciplines over the past three decades by fostering an understanding of risks, benefits, and consequences of available choices and by improving the efficiency and effectiveness of decisional processes.^29^ Donors are increasingly requiring EBDM to merit donor funding. The challenge for disaster professionals is to understand evidence-based processes and integrate them into multi-disciplinary contexts.

## Ascent of Remote Management

Natural disasters have commonly created limitations in access to essential services due to the disruption of telecommunications and transportation links. Complex emergencies have commonly created access limitations due to security breakdown. COVID-19 now is creating access limitations due to movement restrictions as well as epidemic surges in demand at points of care. Remote management techniques, pioneered in disaster settings, are proving increasingly relevant to health management in a pandemic. These techniques include telemedicine, mobile and web communications technology, GPS shipment tracking, remote monitoring, and crowdsourcing of common concerns.

## Population-Based Management

### Historical Perspective

The operational benefit of PBM was first recognized by war surgery practiced in austere environments and illustrated by Coupland, an International Committee of the Red Cross (ICRC; Geneva, Switzerland) surgeon who operated under the harsh environment of the 1992 Afghan War. He practiced PBM when his ICRC hospital infrastructure was disrupted, and vital surgical resources would be wasted on patients whose prognosis was hopeless. This situation underlined the importance of realistic triage for resources when the death rate is unacceptably high among those who should survive. Coupland demonstrated the survival percentage without treatment, survival with treatment, and the possible effect of non-operative management demonstrating that PBM (in wars) might save up to 5%-15% of all casualties.^36^

Whereas PBM has since been applied to additional crises (pandemics and other major disasters), no studies of sufficient quality were identified or published. In 2014, the Task Force Panel for Mass Critical Care reviewed previous recommendations and the literature to develop expert opinion-based suggestions using a modified Delphi process. The Task Force outlined key principles upon which critical care triage should be based, as well as a path for the development of the plans, processes, and infrastructure required. They concluded that ethical and efficient critical care triage is a complex process that requires significant planning and preparation. At present, the prognostic tools required to produce an effective decision support system (triage protocols) as well as the infrastructure, processes, legal protections, and training are largely lacking in most jurisdictions.

During the 2003 US SARS epidemic, individual states varied considerably in their ability to manage the epidemic. State Directors of Health, chronic disease specialists by tradition, were in charge despite little experience or professional interest in disaster management. To remedy this, the CDC offered to send infectious disease epidemiologists to the states, though few took advantage. In 2009, a similar experience gap occurred with H1N1 underscoring the on-going challenges in improving local PH emergency response capabilities. The CDC, recognizing rising concerns of bioterrorism and inadequate state PH preparedness, looked to mutual aid agreements with regional health assets to provide population-based services.^37^ In the early stages of COVID-19, a similar pattern was immediately evident varying widely from state to state resulting in major PH management failures. This resulted in major threats, harassments, and resignations of PH officials, five alone in San Diego (California USA), ceasing any semblance of PH management.^38^

### Operational Imperatives

Lacking a recent pandemic example until now and hindered by a global sense of “protective” denial of its use, the PBM concepts lay dormant or put off to another day. COVID-19 becomes the most recent pandemic example to underscore the critical need for PBM and improved GPH surveillance capacity in the management of Public Health Emergencies of International Concern.^32^ Population-based management decision making in pandemics is a necessary tool for seasoned experts in preventing virus transmission, limiting morbidity and mortality in settings of limited resources, and attenuating the economic and social effects of the pandemic.^39,40^ Burkle emphasized that pandemic PBM experience affirms the need to operationally link prevention, preparation, mitigation, and response to the “surges” that pandemics foster. All pandemics share the following:^40^

- All individuals either have the disease or are susceptible to it;
- All require shared health care needs;
- All require some intervention; and
- Pandemics may require sustained PH operational response lasting 12-24 months (CDC guidelines).

**Figure 1.**
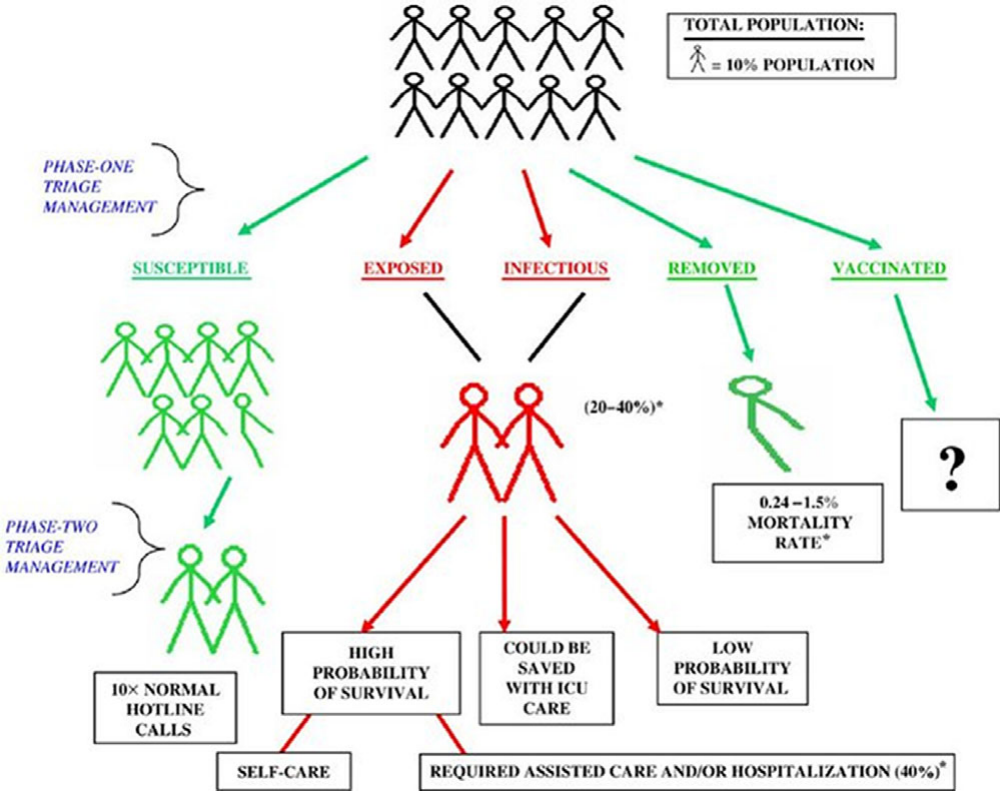
Susceptible-Exposed-Infectious-Removed-Vaccinated (SEIRV) Phase One Triage Categorization. Note: For the entire population and phase two triage management of these subpopulation groups during a bioevent. Percentages based on influenza and SARS outbreak data. Reproduced with permission.^39^ Abbreviations: ICU, intensive care unit; SARS, severe acute respiratory syndrome.

Population-based management data cornerstones are models in three major domains: compartment models (eg, susceptible-exposed-infected-removed-vaccinated [SEIRV]); kinetics models; and decisional models. The cornerstones of PBM are working models in three major technical domains of pandemics: kinetic models or how the virus spreads (eg, incubation period, reproduction ratio, mitigation measure effectiveness);^41^ resource-management and decisional models providing epidemic data and resource availability (eg, capture management awareness of available resources and thresholds for action based on the data emerging from the compartment and kinetic models);^42,43^ and the compartment model used to compute the infected population and the number of casualties of the pandemic, the SEIRV triage model being the most commonly used (Figure 1).^44^

The five PBM SEIRV categories are:^45^

- Susceptible: not exposed, but susceptible;
- Exposed: infected but incubating the virus and are not symptomatic;
- Infectious: contagious;
- Removed: non-contagious and assumed to be “immune” by recovery or non-contagious by death; and
- Vaccinated: a state not available at this time.

The PBM approach to SEIRV-based decision making occurs under a two-phase system with specific measures of effectiveness (MOEs) to increase the likelihood of medical success, epidemic control, and conservation of scarce resources.^35^ Similar to Coupland’s observations, the actual triage management and decision-making practice of PBM in a resource-poor setting is based on:

- Minimal qualifications for survival, which are predetermined agreed-upon criteria on what cases will NOT receive curative care; and
- Exclusion criteria, whereby some medical conditions will not receive the optimal resources they would normally have in non-pandemic conditions.^46^

Unless PBM is fully understood, every operational organization and agency risk increasing, not controlling, transmission rate during a pandemic. If these PBM challenges are not properly addressed, they would become “operational showstoppers.”

In most areas, abundant anecdotal reporting indicates a working relationship between the Emergency Medical Services System (EMSS) and the PH system is lacking. As priorities shift in a pandemic event to hospitals and PH care systems, they need to:

- Improve their capabilities and capacities in surveillance, discovery, and the consequences of different triage and management decisions and interventions in a biothreat environment, starting at the local level;
- Develop triage and management systems (with clear lines of authority) based on PH and epidemiologic requirements, capability, and capacity (triage teams, categories, tags, rapid response, established operational priorities, resource-driven responsible management process), and link local-level surveillance systems with those at the national or regional level;
- Use a triage and management system that reflects the population (cohort) at risk, such as the epidemiologic based SEIRV triage framework; and
- Develop an organizational capacity that uses lateral decision-making skills, prehospital out-patient centers for triage-specific treatments, health information systems, and resource-driven hospital-level predesignated protocols appropriate for a surge of unprecedented proportions. It is recommended that such standards of care should be set at the local to federal levels and spelled out in existing incident-management system protocols.^47,48^

Similar pandemic-related PBM decisions were necessary for hospitals during COVID-19 when critical care equipment was no longer available.^49^ Rosenbaum, facing triage decisions in Italy, emphasized that “first and most important is to separate clinicians providing care from those making triage decisions,” and that a “triage officer, backed by a team with expertise in nursing and respiratory therapy, would make resource-allocation decisions and communicate them to the clinical team, the patient, and the family.” Second, these decisions should be reviewed regularly by a “centralized state-level monitoring committee” (a possible precursor to a PBMT) to ensure that there are no inappropriate inequities. And third, the triage algorithm should also be reviewed regularly as knowledge about the disease evolves.^50^ However, developing the operational relationship between conventional health care and PBM at the time of a pandemic is too late, resulting in a continued vertical relationship, not a lateral one.^50,51^ Indeed, final triage decisions were left to the physician-in-charge in six Northern Italian city hospitals, which future PBMTs would caution might be seen as resulting in an enduring impact on one individual alone which PBMTs attempt to prevent.

Unfortunately, examples of triage-based tragedies during COVID-19 have steadily increased. In Italy, triage guidelines likened the moral choices faced by medical personnel as “wartime triage” criteria resulting in “distributive justice and the appropriate allocation of limited health resources,” maximizing benefits for the largest number through the allocation of an age limit for access to intensive care.^52^ A phased-in criterion uses a “utilitarian approach” where “society, not the physicians, must provide triage guidance that is no longer based on medical priorities alone,” and supports the role of the PBMT’s authority.^53^ Whereas medical triage would be expected as hospital medical resources run low, countries found themselves using a social “risk matrix” to rank COVID-19-positive people outside the hospital setting being ranked as “low, medium, or high” risk. Anyone ranked medium or high were “rejected,” which more often fell to the homeless, foster carers, people needing assistance, and those with mental health conditions who were returned to “unsafe isolation, homelessness, all of which continue to drive disease transmission.”^54^ In the US, race affects one’s ability to flee from a viral “hotspot” to a second safer home, gain access to testing, suffer more frequent treatment delays, are unable to work from home, have increased underlying health conditions, and have poor access to health care (to name but a few), all leading to “extreme social and community disadvantages.”^55^ Without population-based triage management that is universally and consistently applied by experienced PBMTs, there will be unnecessary deaths, moral distress, and lack of public confidence.^56^

Population-based management during a pandemic threat requires a much stronger PH workforce and leadership, acceptance that no one organization or authority possesses all the expertise and resources to manage such an event, and those unprecedented lateral communications, cooperation, knowledge bases, and decision making are detailed in planning documents at all levels.^57^ In the current PBMT model, these decisions would be made within the entire team’s geographic coverage area and supported by community-level data being constantly fed to the GPH Database (Part II), which includes the presence of WHO, national CDC, and PBMTs in every country.

The PBMTs in peaceful times would work collectively to develop a robust, data-driven core capacity of information based on the identification of multiple critical population-based demographic sources required for the PBMT to do their work in prevention, preparedness, response, recovery, and rehabilitation. The PBMTs are formed as interdisciplinary and multi-disciplinary integrative expertise led by PH experts, critical care, infectious disease, epidemiologists, environmental health, and biostatisticians, assisted by available multi-disciplinary expertise in anthropology, sociology, the law, clinical medicine and nursing, pharmacy, industry, and technology who reflect the reality of current field demands, the society served, and health crisis management requirements that define the working relationships and the uncomfortable but real decision making. Health Crisis Managers/Scientists within the PBM framework would receive specialized “disaster cycle” training^58^ (Part II). The stored multiple-population data defines the region and kept in the proposed GPH Database available to each WHO/CDC globally-registered PBMT. The information would include the predictive value of exercise simulations and actual or predictive clinical decisions based on stored data. Every six months, or more when requested, a summary of the data would be forwarded to the WHO, CDC, and PBMTs for review. Data items would focus on information ultimately vital to the decision-making requirements of the PBMTs during a pandemic, such as population densities (future “hot spots”); information vital to immediate implementation of social distancing strategies, ethnocultural and geographic distributions, alternate health resources, staff, and equipment capacities; information vital to triage management decisions and measures of the effectiveness of multiple potential ethnocultural and demographic issues (ie, locations of minority populations) that clearly define the exact locations potentially covered by the PBMT and maximize the PBM decisions.^59,60^ From this information, extensive preventive measures would be launched.

Currently, at the global level, the WHO’s surveillance network collects PH surveillance from all countries, but nationally few data sets can either detect outbreaks or adequately warn relevant PH agencies or the public, underscoring the need for PBMTs supported by a GPH Database (Part II). Additionally, Parrish supports this concept when writing that measuring population health outcomes require: “aggregating outcome measurements made on people, assessing the distribution of individual health outcomes in a population and population subgroups, and measuring the function and well-being of the population, itself, as opposed to individual members.” The latter, he emphasizes, is most critical because it focuses on how well the population produces societal-level conditions that optimally sustain the health of all, not just a few.^61,62^

The PBM models for the large majority of nations would require the expansion of the GPH workforce within the development of the CDC patterned after those in the US, China, and one in Europe, which enjoy direct communication and operational capacity between WHO and country resources. These would build on the existing 86 Field Epidemiology Training Programs (FETPs) infrastructure already serving 160 countries and placed along with other reliable data resources in the proposed GPH Database.^63,64^

**Figure 2.**
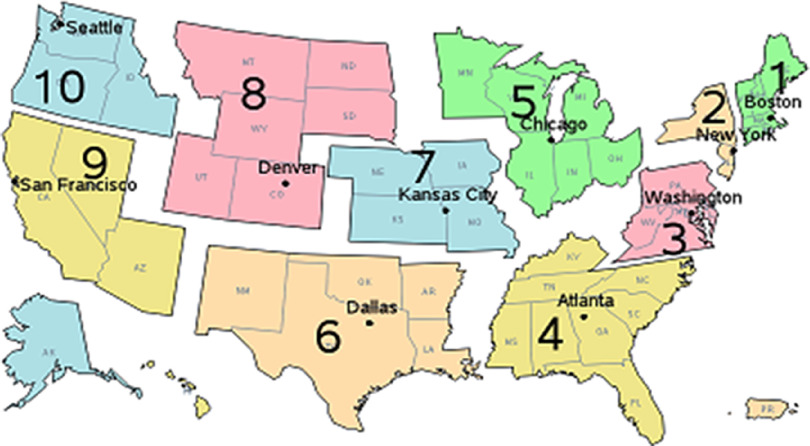
Proposed Restructuring of US HHS/CDC Regional Offices to Sponsor PBMTs. Note: Each of the 10 HHS/CDC regional organizations would house and support a PBMT: PBMTs in the US serve one HHS/CDC regional organization. PBMTs gather and share data with the GPH Database. PBMTs would receive data summaries every six weeks, or more if requested. In normal times, the PBMTs would advise the HHS/CDC regional organization and state health facilities on prevention and preparedness issues gleaned from the GPH Database and both CDC and CDC/HHS regional organizations will assist in propagating the GPH Database with needed information. PBMTs would work with the state and local health departments, hospitals, and clinics, among others, to monitor usage and provide preventive data-based information and timely recommendations. During outbreaks, epidemics, and pandemics, data exchange and summary requests would be daily. If the management situation demands the authority and skillsets of the PBMTs, they will fully engage and manage the pandemic with the legal authority they have. PBMTs are housed in the regional HHS/CDC offices. PBMT permanent staff includes Senior PH Official(s), Medical Expertise (usual infectious diseases/tropical medicine), Biostatisticians, and Epidemiologists, all trained as Disaster Cycle Health Crisis Managers or Scientists. PBMTs are additionally served full- or part-time by a multi-disciplinary team made up of sociologist, anthropologists, and attorneys in International Law. MOE Monitors are members of the PBMTs but are independent. Abbreviations: US, United States; HHS, Health and Human Services; CDC, Centers for Disease Control and Prevention; PBMT, population-based management team; GPH, global public health; PH, public health; MOE, measures of effectiveness.

Population-based management models represent an improved operational arm of the CDC and their designated PBMTs. The number and placement of the PBMTs depend on country-based decisions, primarily focusing on population density and other WHO-country determined risk factors. The operational and functional independence of the 50 independent states in the US provides unique challenges to the proposed PBMT model. Burkle recommends rebuilding and strengthening the HHS/CDC regional organizational offices that directly serve state and local organizations under a Congressionally appointed Regional Director (Figure 2).^16,57^

Under this model, permanent highly trained PBMTs would be placed at each regional office led by PH experts trained in PBM across the entire “Disaster Cycle” of crisis management (prevention, preparedness, response, rehabilitation, recovery)^65^ and assisted by medical experts in infectious diseases and health monitoring, scientists focusing on epidemiology and biostatistics, population-based triage models (SEIRV), viral models and data on anticipated transmission dynamics, decision-making rules (both operational and ethical) for resource-limited settings, and MOEs.

**Figure 3.**
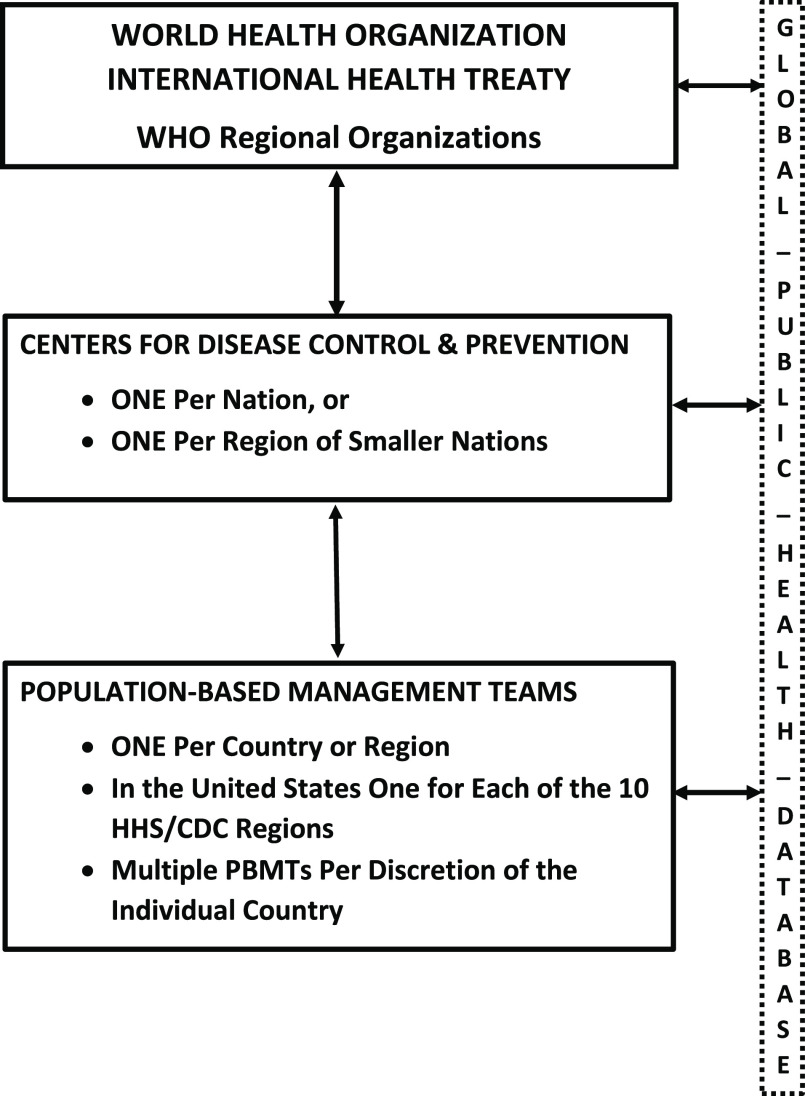
WHO, CDC, and PBMT Users of the GPH Database. Abbreviations: WHO, World Health Organization; CDC, Centers for Disease Control and Prevention; PBMT, population-based management team; GPH, global public health.

These PBMTs would directly serve individual nations, and States within the US HHS/CDC regions, and will have ready access to additional multi-disciplinary experts representing the region’s unique cultural and ethnic qualities of both rural and urban populations, as well as experts in the social sciences and the law. They would be funded by and report directly to the federally independent CDC with operational access to WHO resources, all of which have direct access to the proposed GPH Database. The PBMTs would feed community-level data within their regional jurisdictions that pertain to prevention, preparedness, response, recovery, and rehabilitation of community-level resources. Operationally, PBMTs regularly work with all clinical and governmental resources to both monitor and operationally improve their capacities before, during, and after an outbreak, epidemic, and pandemic. Most importantly, PBMTs have the capacity and authority to assume operational management control if management is beyond local capacity. The decision-making capacity of PBMTs is directly related to data and analytical decision-making capacity of the proposed GPH Database (Part II). To ensure global coverage of PBMTs and support to the GPH data-driven system, the CDC-PBMT model must be replicated in each country, additionally supported by existing WHO Regional Organizations (Figure 3).

## Measures of Effectiveness

Independent MOE monitors are crucial and provide a means for measuring effectiveness, outcome, and performance (including success or failure) of the crisis management. They measure, report, and compare outcomes of the population to improve the population’s experience provided by the PBMT and are sensitive to changes in decisions and operational factors that influence them. The MOEs become essential population-based surveillance assets, especially in a rapidly changing regional environment where they independently speak to the crisis timeline or critical pathways and allow for the horizontal crossing of sector and professional boundaries which may influence both policy decisions and the operationalizing of policy. Traditional use of MOEs are based on international performance but must represent the population-stakeholder, the PBMT, in measuring effectiveness.^66,67^ The MOEs are designed to correspond to accomplishments of mission objectives and achievements of desired results. They quantify the results obtained by a system and may be expressed as probabilities that the system will perform as required. Measuring outcomes, usability efficiency, performance, and suitability are critical. Currently, no clear MOEs exist for determining the success or failure of the management of a pandemic. This is especially critical because management requires multi-agency and multi-disciplinary decision making and evaluation process. It is suggested that the minimum MOEs required to operationally measure outcome must contain a measuring response. The MOE monitors would provide feedback to the entire system, CDC, and PBMTs. In the proposed US model, replicated globally, MOE information would go directly to HHS/CDC Regional Offices and their PBMTs where their data and recommendations would be included in the proposed GPH Database infrastructure for immediate action (Part II).

“*Denial is the strongest of human defense mechanisms and one used often when faced with seemingly impossible challenges.*” *Burkle FM, 1992*
